# Discovery and Diagnosis of a New Sobemovirus Infecting *Cyperus esculentus* Showing Leaf Yellow Mosaic and Dwarfism Using Small-RNA High Throughput Sequencing

**DOI:** 10.3390/plants11152002

**Published:** 2022-07-31

**Authors:** Luis Rubio, Francisco J. J. Guinot-Moreno, Carmen Sanz-López, Luis Galipienso

**Affiliations:** 1 Instituto Valenciano de Investigaciones Agrarias (IVIA), 46113 Moncada, Valencia, Spain; lrubio@ivia.es (L.R.); jguinot97@gmail.com (F.J.J.G.-M.); carmesanz@gmail.com (C.S.-L.); 2 Universidad Católica de Valencia “San Vicente Mártir”, 46002 Valencia, Valencia, Spain; 3 Universitat Politècnica de València, 46022 Valencia, Valencia, Spain

**Keywords:** tiger nut, chufa, RT-qPCR, RT-LAMP

## Abstract

*C. esculentus* is a profitable crop in Valencia, Spain, but the emergence of a disease causing of leaf yellow mosaic, dwarfism, and a drastic decrease in tuber production has become a problem. The small-RNA high-throughput sequencing (HTS) of a diseased *C. esculentus* plant identified only one virus, which could be the causal agent of this disease. The amino-acid comparison with viral sequences from GenBank and phylogenetic analyses indicated that this was a new species of genus *Sobemovirus,* and the name *Xufa yellow dwarf virus* was proposed. Completion with Sanger sequencing yielded a contig of 3072 nt corresponding to about 75% of the typical genome of sobemoviruses, including ORFs 2a (polyprotein-containing protease, VPG, and other proteins), 2b (RNA-dependent RNA polymerase), and 3 (coat protein). The nucleotide sequence was used to develop fast and accurate methods for the detection and quantification of xufa yellow dwarf virus (XYDV) based on reverse transcription (RT) and DNA amplification. XYDV was detected in leaves and tubers and showed a high incidence in the field in both symptomatic (almost 100%) and asymptomatic (70%) plants, but its accumulation was much higher in symptomatic plants. The relevance of these results for disease control was discussed.

## 1. Introduction

*Cyperus esculentus*, known as tiger nut, chufa or xufa, yellow nutsedge, iron water chestnut, underground chestnut, underground walnut, earth almond, ginseng fruit, ginseng bean, etc., is considered an invasive weed in most tropical, subtropical, and Mediterranean regions. However, *C. esculentus* is cultivated in a few places, since it produces edible sweet nut-like tubers, which are used mainly as a side dish in some Western African countries and as fodder for animals in some American countries [1]. In Europe, *C. esculentus* is cultivated mainly in a small region of about 400 ha in Valencia, Spain, and is used to elaborate a sweet milky beverage named horchata that is served cold in summer as a natural refreshment. The elaboration and commercialization of horchata represent a very profitable industry in Valencia, producing about 50 million liters per year that are prepared from about 6000 tons of dried tubers, where half of them is produced in Valencia and the other half is imported from Western Africa.

**Figure 1:**
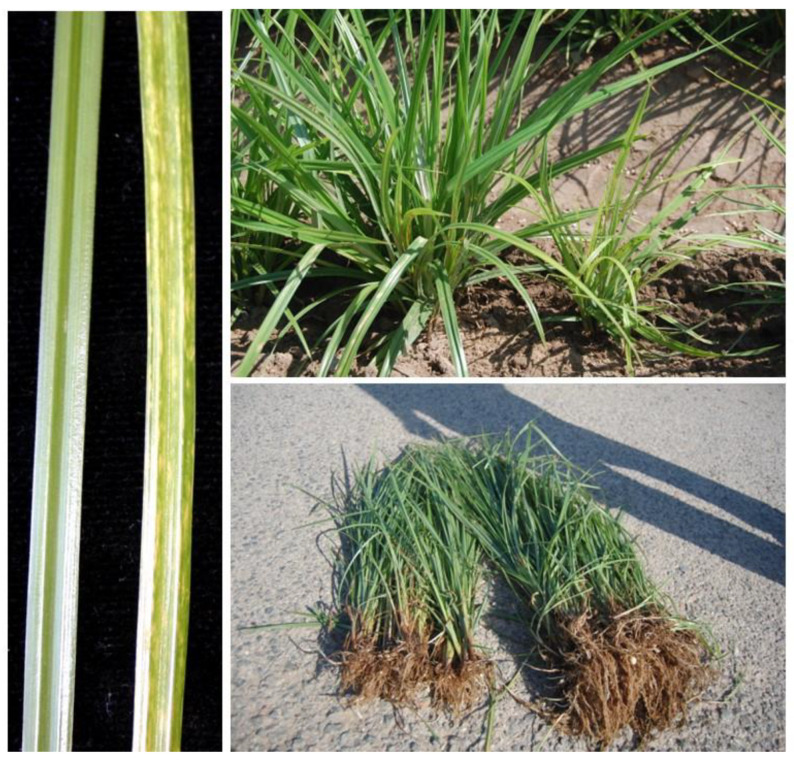
Symptoms found in some *C. esculentus* plants: leaves with yellowing or chlorosis following a mosaic pattern in streaks and stunting in the aerial parts and roots with lower tuber production.

Since 2015, farmers have found diseased plants showing leaves with a yellowed mosaic pattern of parallel discontinuous streaks, dwarfism in the aerial parts and roots, and a severe reduction in tuber production (Figure 1).

The first step to eradicate or mitigate the disease is the identification of the causal agent. The symptoms of leaf yellowing and plant stunting present in these *C. esculentus* plants are typical of viral infections. The high-throughput sequencing (HTS) of viral small RNAs (produced by the plant defense mechanism based on RNA silencing) is a powerful tool generating in a short time thousands of unbiased sequences corresponding to all viruses present in the sample [2,3]. HTS does not require previous knowledge of the targeted organisms, and it is especially suited to discovering new viruses. The obtained nucleotide sequences can be ascribed to different taxonomic levels, such as virus species, genera, o families, according to the nucleotide or amino-acid identity with known viral sequences from the GenBank database or the presence of sequence motifs [4].

However, HTS is too expensive for routine diagnosis, and it is necessary to develop rapid and accurate detection techniques for each virus to study virus incidence and epidemiology and apply disease-control measures such as certification and eradication. The techniques based on reverse transcription (RT) and DNA amplification are the fastest to be developed and can be designed from the sequences obtained with HTS. Polymerase chain reaction (PCR) is the most widely used, enabling fast, highly specific, and sensitive diagnosis, but real-time quantitative PCR (qPCR) with SYBR Green or TaqMan probes is more sensitive and can quantify the virus titer, which is very useful to evaluate disease-control measures such as the plant breeding of resistant cultivars and cross protection [4]. Loop-mediated isothermal amplification (LAMP) is a specific and sensitive technique with the advantage of not requiring expensive thermal-cycling instruments, being less sensitive to inhibitors, unlike PCR and qPCR, and having a great potential to be used directly on field [5].

In this work, a *C. esculentus* plant with leaf mosaic yellowing and dwarfism was analyzed using small-RNA HTS, showing that only one virus was present. The amino-acid sequence comparison and phylogenetic analyses showed that it was a new species of genus *Sobemovirus* that was proposed to be named *Xufa yellow dwarf virus*. The obtained nucleotide sequence was used to develop RT-PCR, RT-PCR with SYBR and a TaqMan probe, and RT-LAMP for the fast and specific detection and quantification of xufa yellow dwarf virus (XYDV). These detection methods were applied to detect XYDV in leaves and tubers, evaluate its incidence on field and estimate XYDV accumulation in plants.

## 2. Results

### 2.1. Identification of a New Sobemovirus

**Figure 2:**
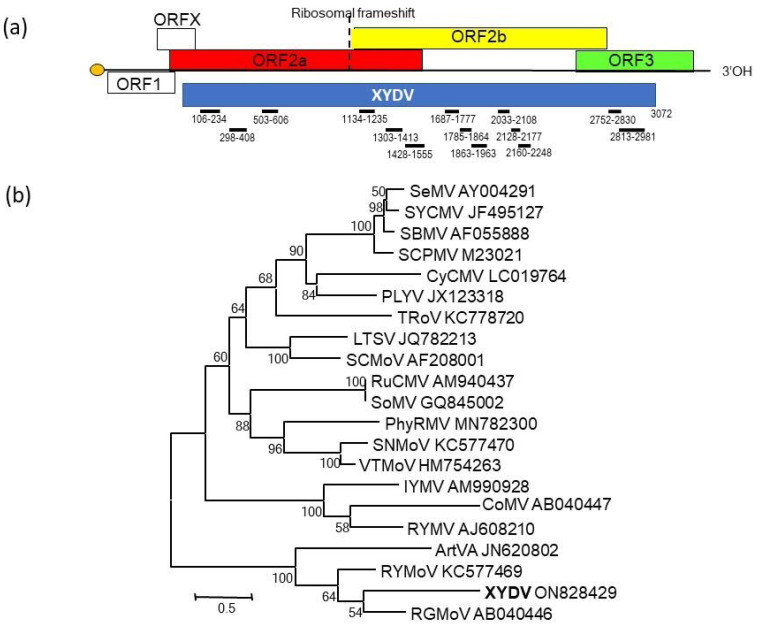
(**a**) Genome organization of sobemoviruses [7] and the sequenced genome of xufa yellow dwarf virus (XYDV) with the contigs obtained with HTS shown below. (**b**) Unrooted Maximum Likelihood phylogenetic tree of coat-protein amino-acid sequences of members of genus *Sobemovirus* (Table 2) and XYDV. GenBank accession numbers and significant bootstrap values are indicated.

The HTS of RNA extracts from a *C. esculentus* diseased plant yielded 622 contigs, but only 15 corresponded to viruses or viroids, and the rest were from the host. The Blastn analyses showed a 59 nt contig with a 92% identity with Hop stunt viroid (HSVd). However, the real-time RT-qPCR analysis with specific HSVd primers [6] was negative for this plant and other plants with the same symptoms. In addition, the 59 nt contig was formed by small RNAs with low reads and no similarity with HSVd, indicating that HSVd was not present in these plants and that this contig was an assembly artifact. The Blastx analyses showed 14 contigs with amino-acid similarity with viruses of genus *Sobemovirus,* such as rottboelia yellow mottle virus (RYMoV) and ryegrass mottle virus (RGMoV). To fill the gaps between contigs and confirm the sequences obtained with HTS, primers were designed (Table 1) and used for RT-PCR and Sanger sequencing. The sequence was extended by 105 and 91 nt towards the 5′ and 3′ ends, respectively, with a 5′/3′ RACE system. When all sequences were assembled, a single contig of 3072 nt was obtained; this corresponded to the genome of a virus with amino-acid similarity with members of genus *Sobemovirus* (Figure 2a). This virus was named *Xufa yellow dwarf virus* based on *C. esculentus’* name in the local language, “xufa” (Valencian/Catalan), and the symptoms. No other viruses or viroids were identified.

About 75% of the genome of xufa yellow dwarf virus (XYDV) was sequenced and showed the typical genomic organization of the genus *Sobemovirus* [7]. The XYDV sequence included three ORFs (Figure 2a). ORF 2a (about 90% sequenced) encodes polyprotein P2a containing a transmembrane-anchoring domain, a serine protease, a VPg, and other products called P10 and P8. ORF 2b (about 94% sequenced) encodes an RNA polymerase RNA dependent (RdRp) expressed as a fusion protein via a −1 programmed ribosomal frameshift (−1PRF) with respect to ORF 2a. The XYDV sequence contains the −1 PRF signal consisting of a slippery sequence, 5′-UUUAAAC-3′, followed by a stem-loop structure. ORF 3 (about 75% sequenced) encodes the coat protein (CP) expressed by a subgenomic RNA. This nucleotide sequence was deposited in GenBank under accession number ON828429.

The comparison of the XYDV sequence with those of the members of genus *Sobemovirus* showed amino-acid identities ranging from 10.8 to 63.2% (Table 2). The most similar viruses to XYDV were RYMoV, RGMoV, and artemisia virus A (ArtVA). The three ORFs showed different levels of variability, with ORF 2b (RdRp) being the most conserved and ORF 3 (CP) the most variable. The phylogenetic relationships were similar for the three ORFs, so only ORF 3 (CP) is shown in Figure 2b. XYDV formed a statistically supported clade with RGMoV, RyMoV, and ArtVA, confirming that XYDV is a member of genus *Sobemovirus*, and the low amino-acid identity between these viruses indicates that XYDV is a different species of this genus.

### 2.2. Development of Methods for Fast Detection and Quantification of XYDV

Based on the obtained nucleotide sequence, primers and a TaqMan probe (Table 1). were designed for techniques based on reverse transcription (RT) and DNA amplification: polymerase chain reaction (PCR), real-time quantitative PCR (qPCR) with SYBR Green, qPCR with a TaqMan probe, and loop-mediated isothermal amplification (LAMP). These techniques detected XYDV in the leaves and tubers of *C. esculentus* plants and were negative for the negative controls, which were water, RNA and crude extracts from healthy wild *C. esculentus* plants and *Nicotiana benthamiana* plants and serial dilutions from these extracts (data not shown).

**Figure 3:**
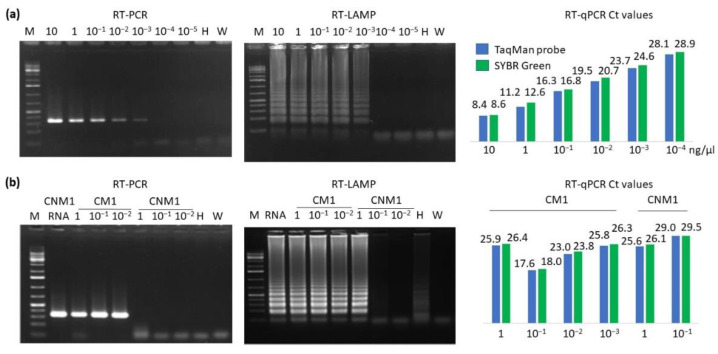
Evaluation of XYDV detection techniques: (**a**) sensitivity assessed using serial dilutions of 10 ng/µL RNA extracts; (**b**) serial dilutions of plant crude extracts from two XYDV isolates, CM1 and CNM1. CMN1 RNA was used as an RT-PCR positive control. M = GeneRuler 1 kb plus ladder (Thermo Fisher Scientific, Waltham, MA, USA). Negative controls: H = RNA or crude extracts from *C. esculentus* healthy plants and W = water. No amplifications were obtained with RT-qPCR of the negative controls.

The sensitivity of these techniques was compared using serial ten-fold dilutions of 10 ng/µL total-RNA extracts from an XYDV-infected plant (Figure 3a). RT-PCR and RT-LAMP detected XYDV until the fourth dilution, corresponding to 1 pg, whereas RT-qPCR with SYBR or the TaqMan probe detected it in the fifth dilution, corresponding to 100 fg. The Ct values were similar for both RT-qPCR techniques, being slightly lower for RT-qPCR with the TaqMan probe (Figure 3a). No amplifications were obtained from the negative controls.

To find out if the RNA-extraction step could be avoided, these amplification methods were assayed with concentrated and ten-fold serial dilutions of crude plant extracts (only ground with buffer) from two XYDV isolates: CM1, obtained from a plant with severe symptoms, and CMN1, from an asymptomatic plant (hypothesized having lower viral titer; see below). RT-PCR detected XYDV in the CM1 crude-extract concentrate and the two subsequent 10-fold dilutions but not in any of the CMN1 crude extracts, despite being positive for the RNA extracts (Figure 3b). The intensity of the RT-PCR bands for CM1 was similar for the crude extract and the three serial dilutions, suggesting that the viral RNA titer at the lowest dilution was enough to attain maximum amplification. In addition, the concentrated crude extracts did not contain enough inhibitors to interfere with RT-PCR.

RT-LAMP detected XYDV in the CM1 concentrate and the two dilutions, as well as the CMN1 concentrate. Finally, RT-qPCR with SYBR Green and the TaqMan probe detected XYDV in CM1 crude extracts until dilution 1/1000 and CMN1 until dilution 1/10 (Figure 3b). Curiously, the Cts of the CM1 concentrate were higher than those of dilutions 1/10 and 1/100, suggesting the presence of inhibitors.

### 2.3. Incidence of XYDV in the Field and Viral Accumulation

**Figure 4:**
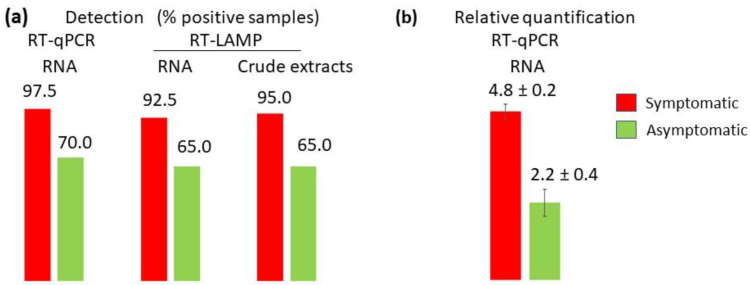
Analysis of leaves from symptomatic and asymptomatic *C. esculentus* plants collected in the field: (**a**) percentage of positive samples; (**b**) relative quantification expressed in a logarithm scale.

Leaf samples were collected from 40 symptomatic and 20 asymptomatic *C. esculentus* plants from eight plots of the five most productive municipalities of Horta Nord comarca (county), Valencia province, Spain, in 2019. Crude extracts were analyzed with RT-LAMP, and RNA extracts were analyzed with RT-qPCR using the TaqMan probe and with RT-LAMP (Figure 4a). The RT-qPCR analysis of RNA extracts detected XYDV in 39 symptomatic plants (97.5 %), whereas RT-LAMP was a little less sensitive and detected XYDV in 37 or 38 (92.5 or 95.5%) symptomatic-plant RNA or crude extracts, respectively. Curiously, XYDV was also detected in a high proportion of asymptomatic plants (70.0% with RT-qPCR). The difference in XYDV incidence between symptomatic and asymptomatic plants was not statistically significant. XYDV was also detected in the tubers from five symptomatic plants with RT-LAMP and RT-qPCR.

The RT-qPCR analyses showed a correlation between XYDV accumulation in leaves and symptom manifestation, since the XYDV titer was about 100 times higher in symptomatic (4.8 ± 0.2) than in asymptomatic plants (2.2 ± 0.4), and this difference was statistically significant (Figure 4b). The accumulation of XYDV in tubers from the five symptomatic plants was high (4.2 ± 0.3), although it was significantly higher in the leaves of these plants (5.4 ± 0.1).

## 3. Discussion

The cultivation of *C. esculentus* and the elaboration of horchata have become substantial revenue sources for the county of Horta Nord in Valencia, Spain. However, some diseases have risen in the last decades, such as tuber rot [8] and leaf necrosis [9] produced by fungi and the black spot of unknown etiology [10]. These diseases have caused severe economic losses and threatened tuber production. This work described the first viral disease detected in *C. esculentus* in Spain. Small-RNA HTS enabled the identification of a new virus of genus *Sobemovirus,* and the name *Xufa yellow dwarf virus* was proposed. To our knowledge, only four viruses infecting *C. esculentus* have been reported: turnip mosaic virus (TuMV) in Zimbabwe [11]; impatiens necrotic spot virus (INSV) in Georgia, USA [12]; brome streak mosaic virus (BrSMV) in Hungary [13]; and rice yellow mottle virus (RYMV) in Nigeria [14]. The low number of viruses described worldwide is probably due to having been poorly investigated, since *C. esculentus* is not widely used in agriculture. The emergence of viral diseases in different crops in Valencia and other Mediterranean areas is frequent, mainly caused by intensive agriculture, climate change, and the global movement of plant material [15].

The detection of xufa yellow dwarf virus (XYDV) in tubers could be important, since tubers are used as seeds and could be a means of transmission, as reported for some sobemoviruses [16]. Thus, XYDV could have been introduced in Valencia by the importation of *C. esculentus* tubers from Western Africa. Tubers are imported without a phytosanitary passport, as they can be only used for human or animal consumption. However, some farmers cultivate African tubers despite this being forbidden, with a high risk of dispersing pathogens and pests. Curiously, another sobemovirus, RYMV, has been also detected in *C. esculentus* in Western Africa [14]. Knowing the transmission means of viruses is key to applying prophylactic measures for disease control, e.g., the certification and control of vectors. Transmission assays and/or epidemiological studies are necessary to know if XYDV is transmitted by tubers, mechanical wounding, and/or insects as other sobemoviruses [16]. In case XYDV is tuber-transmitted, in addition to the sanitary certification of imported tubers, other control measures should be tested, such as the selection of XYDV-free tubers, chemical and/or thermal disinfection, or in vitro culture [4,10]. The molecular techniques developed here are very sensitive and can be used to test the effectiveness of removing XYDV from tubers. RT-LAMP could be used by farmers in situ; since it only requires a thermal bath, it can be directly used with plant crude extracts, and the amplification can also be visualized by means of naked-eye inspection using turbidity-changing ion indicators or color-changing fluorescent molecules [5].

These techniques were applied in the survey of a field, and XYDV was detected in almost all *C. esculentus* plants with leaf yellow mosaic and dwarfism and in 70% of the asymptomatic plants. The presence of XYDV in asymptomatic plants and the high overall incidence of this virus do not make the use of eradication strategies such as roguing feasible [4]. In this scenario, the most plausible disease-control strategy would be using virus-free tubers as seeds. Farmers could test the presence of XYDV in these tubers with RT-LAMP in each sowing season and evaluate the incidence in the field. However, as mentioned above, it is necessary to know if XYDV can be transmitted by other means, which would require additional measures. The RT-qPCR analyses showed that the XYDV titer was significantly much higher in symptomatic than in asymptomatic plants. This suggests that XYDV could interfere with root development and plant growth in seedlings when XYCV concentration surpasses a certain threshold. A positive correlation between symptoms and viral accumulation has been also reported for several plant viruses [17,18,19], although this does not apply to tolerant hosts [20]. The correlation among XYDV accumulation and symptom manifestation, as well as the fact that XYDV was the only viral sequence found using HTS strongly supports that XYDV is the causal agent of the disease of leaf yellow mosaic and dwarfism in *C. esculentus*.

## 4. Materials and Methods

### 4.1. Plant Material

Leaf samples and tubers were collected from 40 symptomatic and 20 asymptomatic *C. esculentus* plants from eight plots in the municipalities of Alboraya, Burjassot, Godella, Massalfasar, Moncada, and Vinalesa, which belong to the comarca (county) of Horta Nord, Valencia province, Spain.

### 4.2. Extract Preparation

About 200 mg of leaf tissue or tuber was ground with liquid nitrogen in microtubes containing glass beads in a TissueLyser power homogenizer (Qiagen, Hilden, Germany). Total-RNA extracts were obtained with Spectrum Plant Total RNA Kit (Sigma-Aldrich, San Luis, MO, USA). Crude extracts were prepared by means of the resuspension of ground plant tissue in 500 µL of STE buffer (100 mM NaCl, 10 mM Tris-HCl,1 mM EDTA (pH 7.5)) and homogenization with a vortex mixer for 2 min. RNA and crude extracts were kept at −80 °C until use.

### 4.3. Sequencing

For the HTS of small RNAs, total-RNA concentration and purity were determined with the Qubit^®^ RNA assay kit in a Qubit^®^ 3.0 fluorometer (Thermo Fisher Scientific, Waltham, MA, USA) and a NanoPhotometer^®^ spectrophotometer (Implen, Westlake Village, CA, USA), respectively. RNA integrity was determined in an Agilent Bioanalyzer 2100 system with the RNA Nano 6000 assay kit (Agilent Technologies, Santa Clara, CA, USA). cDNA was obtained from 1 µg of total-RNA with NEBNext^®^ Multiplex Small RNA Library Prep Set for Illumina^®^ (Sigma-Aldrich, San Luis, MO, USA) and sequenced in the Illumina NextSeq550 platform (Illumina, San Diego, CA, USA). Sequencing adapters were trimmed from the reads, and low-quality reads were filtered in November 2019 using SeqTrimNext V2.0.67 software (https://github.com/dariogf/SeqtrimNext, accessed on 30 November 2019) by applying the standard parameters for Illumina short reads [21]. VirusDetect V 1.7 [22] and the virus nucleotide database (http://bioinfo.bti.cornell.edu/ftp/program/VirusDetect/virus_database/v239, accessed on 30 November 2019) were used for virus identification. Reads were aligned with the virus nucleotide database with the Burrows–Wheler Aligner program [23] and assembled with Velvet V.1.2.09 software [24]. Reads not aligning with viral sequences were recovered, assembled, and compared to the GenBank database with Blastn and Blastx [25] to find similar viral nucleotide and/or protein sequences. Sanger sequencing was performed on RT-PCR products purified using QIAquick PCR Purification Kit (Qiagen) using a Big Dye Terminator V. 3.0 Cycle Sequencing kit (Thermo Fisher Scientific, Waltham, MA, USA) in an ABI 3130 XL capillary sequencer (Applied Biosystems, Thermo Fisher Scientific, Waltham, MA, USA). Afterwards, 5′/3′ RACE Kit, 2nd Generation (Roche, Basilea, Switzerland), was used to determine the 5′ and 3′ terminal sequences.

### 4.4. Amino-Acid Identity and Phylogenetic Analyses

The nucleotide sequences of ORFs 2a, 2b, and 3 of XYDV were translated to amino acids (https://web.expasy.org/translate, accessed on 30 November 2019), and the equivalent sequences of members of genus *Sobemovirus* were retrieved from GenBank (Table 2). These sequences were aligned with the CLUSTALW algorithm [26] implemented in MEGA X software [27]. Amino-acid identities were estimated with formula 100 × (1 − pdistance) in MEGA X. The phylogenetic relationships of the three ORFs were inferred with the Maximum Likelihood method [28] using the LG + F + G model, which is based on the LG matrix of amino-acid substitution [29] with empirical amino-acid frequencies and five discrete gamma rates. The statistical significance of the internal nodes was estimated with a bootstrap analysis with 100 replicates [30]. All these analyses were carried out in MEGA X.

### 4.5. Primer and Probe Design

The nucleotide sequence obtained for XYDV was used to design primers for RT-PCR and RT-qPCR and a TaqMan probe with Primer3 [31] and Primer Express (Thermo Fisher Scientific, Waltham, MA, USA). Primers for RT-LAMP were designed with LAMP Designer 1.16 (Premier Biosoft, Palo Alto, CA, USA). The list of the designed primers is shown in Table 1.

### 4.6. RT and DNA Amplification

RT was performed by denaturing total RNA or plant crude extracts mixed with 5 µM of random hexamers by heating at 65 °C for 5 min and incubating on ice for 1 min; then, they were added to a 20 µL reaction mixture containing 5 mM DTT, 1 mM of each dNTP, first-strand buffer, 20 units of RNaseOUT™ RNase Inhibitor (Invitrogen), and 100 units of SuperScript IV Reverse Transcriptase (Invitrogen, Thermo Fisher Scientific, Waltham, MA, USA). The reaction mixture was incubated at 23 °C for 10 min, at 50 °C for 10 min, and at 80 °C for 10 min to inactivate the reaction.

PCR was performed in a 20 µL reaction mixture containing PCR buffer, 1.5 mM MgCl_2_, 1 mM of each dNTP, 1 µM of primers X1F and X1R (Table 1), and 0.5 U of Taq DNA polymerase (Invitrogen, Thermo Fisher Scientific, Waltham, MA, USA). Eppendorf^®^ Mastercycler EP Gradient was used under the following thermocycling conditions: denaturation at 94 °C for 2 min; 40 cycles at 94 °C for 30 s, at 52 °C for 1 min, and at 72 °C for 45 s; and an extension step at 72 °C for 5 min. PCR products were analyzed by means of electrophoresis in 2% agarose gels and visualized with Gel Red (Biotium. Fremont, CA, USA) under UV light.

For qPCR, total-RNA concentration was measured with a NanoDrop™ 1000 spectrophotometer and adjusted to 10 ng/µL to normalize the different extractions. Real-time qPCR with SYBR Green was carried out in LightCycler^®^480 (Roche Molecular Systems Inc.) in a 20 µL mixture containing TB Green Premix Ex Taq II (Tli RNase H Plus) (Takara), 0.2 µM of primers qX1F and qX1R (Table 1), and 1/10 of cDNA from the RT reaction. The thermocycling conditions included denaturation at 95 °C for 30 s and 35 cycles of 95 °C for 5 s and at 60 °C for 30 s. The melting-curve analysis of the PCR products showed only one peak, indicating the absence of nonspecific products and/or primer-dimers. One-step real-time RT-qPCR with a TaqMan probe was performed in LightCycler^®^480 (Roche Molecular Systems, Pleasanton, CA, USA) in a 20 µL mixture prepared with One Step PrimeScript RT-PCR Kit (TaKaRa, Shiga, Japan ) and containing 0.2 µM of primers qX2F and X2R and 0.4 µM TaqMan probe (Table 1). The thermocycling conditions consisted of RT at 42 °C for 25 min, followed by denaturation at 95 °C for 15 s and 35 cycles of 95 °C for 5 s and at 60 °C for 20 s. For the relative quantification of XYDV, a standard curve was obtained with the RT-qPCR of the sample with the lowest Ct and 10-fold serial dilutions assigning arbitrary values starting from seven. The standard curve showed a high determination coefficient (R^2^ = 0.9994), indicating a strong linear relationship, and the linear regression formula was y = −3.642x + 27.02.

One-step RT-LAMP was performed in a 25 µL mixture containing a sample (RNA of crude extracts) previously denatured at 95 °C for 5 min, Isotermal Amplification Buffer, 1.4 mM dNTPS, 5 U WarmStar RT (New England Biolabs, Ipswich, MA, USA), 8 U Bst DNA polymerase (New England Biolabs, Ipswich, MA, USA), and the three XYDV-specific primer pairs: 0.2 µ XF3 and XB3, 1.6 µM XFIP and XBIP, and 0.4 µM XLoopF and XLoopR (Table 1). The mixture was incubated at 68 °C for 1 h in a water bath and heated to 80 °C for 10 min to stop the reaction. The amplification products were analyzed by means of electrophoresis in 2% agarose gels.

### 4.7. Statistical Analysis

Symptomatic and asymptomatic plants were compared for XYDV incidence and accumulation with chi-squared and unpaired two-tailed Student’s t-tests, respectively. The analyses were performed with Statgraphics plus Software Version 5.1 using an alpha level of 0.05. 

## Figures and Tables

**Table 1:** Oligonucleotides (primers and TaqMan probe) designed and used in this work.

Use	Oligo	Sequence
RT-PCR and Sanger	479F	5′-AAGATGTGATCCTCCAGCC-3′
sequencing	402R	5′-CAGCTTGGACCAGACAGAA-3′
	541R	5′-GGGTATATCTAGCGAAGT-3′
	253F	5′-TCAAATTTAGAGAGTCTGGTCG-3′
	253R	5′-CAGACTCTCTAAATTTGAC-3′
	171F	5′-AGGACGATTCCGCTTGATATC-3′
	171R	5′-ATATCAAGCGGAATCGTCCTTC-3′
	148R	5′-TGCAGTACGATCCAGATTTC-3′
	220F	5′-GTTAAACTTTAACGCTAGGAATG-3′
RT-PCR	X1F	5′-ACGACTTAGTCGTTGAAGC-3′
	X1R	5′-TCCGCGTATTCCCAGATAGC-3′
RT-qPCR	qX1F	ACGTGCTTGATGCCGCTAAG
(SYBR Green)	qX1R	GGAACCTGTACCGCGGAGAT
RT-qPCR	qX2F	5′-GTGCAATGCGGGAAATCC-3′
(TaqMan probe)	qX2R	5′-AGCTTAGCGGCATCAAGCA-3′
	Xprobe	5′Fam-CCGTGTTGCTCACAGCATGGCA-Tamra3′
RT-LAMP	XF3	5′-TTCCACCGTCTCCTACAG-3′
	XB3	5′-TCCACACCTGCGTATGTA-3′
	XFIP	5′-GCGGAGAAGAATCTCACTCGGTGAACTTCAGTGGCTTGC-3′
	XBIP	5′-AGTGGCGACATTGCGATAGGCTGGTATGGTATGGTGACTGTAGTTG-3′
	XLoopF	5′-CTCGGAACTTCTGGTATCTCTG-3′
	XLoopR	5′-GTTGTGTATGACTCCGCTGA-3′

**Table 2:** Amino-acid identities between xufa yellow dwarf virus (XYDV) and viruses of genus *Sobemovirus* for ORFs 2a, 2b, and 3.

Virus	Acronym	GenBank	2a	2b	3
Artemisia virus A	ArtVA	JN620802	32.5	58.3	24.4
Cocksfoot mottle virus	CoMV	AB040447	25.1	51.3	11.0
Cymbidium chlorotic mosaic virus	CyCMV	LC019764	28.6	51.4	20.5
Imperata yellow mottle virus	IYMV	AM990928	21.1	51.3	10.8
Lucerne transient streak virus	LTSV	JQ782213	26.7	54.5	17.6
Papaya lethal yellowing virus	PLYV	JX123318	26.7	53.3	16.3
Physalis rugose mosaic virus	PhyRMV	MN782300	27.7	51.3	16.1
Rice yellow mottle virus	RYMV	AJ608210	26.1	53.6	17.6
Rottboellia yellow mottle virus	RYMoV	KC577469	40.6	63.2	33.7
Rubus chlorotic mottle virus	RuCMV	AM940437	26.0	52.3	18.1
Ryegrass mottle virus	RGMoV	EF091714	39.6	61.1	35.1
Sesbania mosaic virus	SeMV	AY004291	27.0	50.8	20.7
Solanum nodiflorum mottle virus	SNMoV	KC577470	26.0	48.7	20.7
Southern bean mosaic virus	SBMV	DQ875594	25.0	51.8	21.6
Southern cowpea mosaic virus	SCPMV	NC_001625	27.7	52.8	19.6
Sowbane mosaic virus	SoMV	GQ845002	26.0	51.9	19.4
Soybean yellow common mosaic virus	SYCMV	JF495127	26.5	50.4	22.0
Subterranean clover mottle virus	SCMoV	AF208001	27.7	54.9	18.0
Turnip rosette virus	TRoV	KC778720	29.4	50.0	16.6
Velvet tobacco mottle virus	VTMoV	HM754263	27.2	48.7	21.0

## Data Availability

The nucleotide sequence of the virus with the proposed name xufa yellow dwarf virus generated in this work was deposited in GenBank under accession number ON828429. All the sequencing-output datasets generated in the study are freely available upon request to the corresponding author.
